# Risk Factors of Esophageal Squamous Cell Carcinoma beyond Alcohol and Smoking

**DOI:** 10.3390/cancers13051009

**Published:** 2021-02-28

**Authors:** Munir Tarazi, Swathikan Chidambaram, Sheraz R. Markar

**Affiliations:** 1 Department of Surgery and Cancer, Imperial College London, London W2 1NY, UK; munirtarazi@rcsi.ie (M.T.); swathikan.chidambaram12@imperial.ac.uk (S.C.); 2 Department of Molecular Medicine and Surgery, Karolinska Institutet, Karolinska University Hospital, 17164 Stockholm, Sweden

**Keywords:** risk factor, squamous cell carcinoma, SCC, esophageal

## Abstract

**Simple Summary:**

Esophageal squamous cell carcinoma (ESCC) is the sixth most common cause of death worldwide. Incidence rates vary internationally, with the highest rates found in Southern and Eastern Africa, and central Asia. Initial studies identified multiple factors associated with an increased risk of ESCC, with subsequent work then focused on developing plausible biological mechanistic associations. The aim of this review is to summarize the role of risk factors in the development of ESCC and propose future directions for further research. A systematic literature search was conducted to identify risk factors associated with the development of ESCC. Risk factors were divided into seven subcategories: genetic, dietary and nutrition, gastric atrophy, infection and microbiome, metabolic, epidemiological and environmental, and other risk factors. Risk factors from each subcategory were summarized and explored. This review highlights several current risk factors of ESCC. Further research to validate these results and their effects on tumor biology is necessary.

**Abstract:**

Esophageal squamous cell carcinoma (ESCC) is the sixth most common cause of death worldwide. Incidence rates vary internationally, with the highest rates found in Southern and Eastern Africa, and central Asia. Initial observational studies identified multiple factors associated with an increased risk of ESCC, with subsequent work then focused on developing plausible biological mechanistic associations. The aim of this review is to summarize the role of risk factors in the development of ESCC and propose future directions for further research. A systematic search of the literature was conducted by screening EMBASE, MEDLINE/PubMed, and CENTRAL for relevant publications. In total, 73 studies were included that sought to identify risk factors associated with the development of esophageal squamous cell carcinoma. Risk factors were divided into seven subcategories: genetic, dietary and nutrition, gastric atrophy, infection and microbiome, metabolic, epidemiological and environmental and other risk factors. Risk factors from each subcategory were summarized and explored with mechanistic explanations for these associations. This review highlights several current risk factors of ESCC. These risk factors were explored, and explanations dissected. Most studies focused on investigating genetic and dietary and nutritional factors, whereas this review identified other potential risk factors that have yet to be fully explored. Furthermore, there is a lack of literature on the association of these risk factors with tumor factors and disease prognosis. Further research to validate these results and their effects on tumor biology is absolutely necessary.

## 1. Introduction

Squamous cell carcinoma of the esophagus (ESCC) is an aggressive condition that ranks seventh in incidence and sixth in mortality among all malignancies worldwide. It is the more prevalent histological variant of the two main types of esophageal cancers. There is a clear geographic variation in the distribution of ESCC globally. The prevalence is higher in east Asia, eastern and southern Africa, as well as southern Europe, while it is lower in North America and other parts of Europe. This geographical variation is clearly demonstrated as the annual incidence in the United States is 3.5/100,000 persons, while it is significantly higher at 115/100,000 in countries such as Iran. Such epidemiological differences have led to an evaluation of specific risk factors that predispose patients to the development of ESCC. Initial observational studies identified lifestyle factors such as diet, smoking, and obesity to be associated with an increased risk of ESCC. Subsequent work then developed mechanisms to explain these relationships at a molecular level, which subsequently led to further elaboration of other less obvious yet more permeating risk factors. This review aims to summarize the role of risk factors in the development of ESCC and propose areas of future research.

## 2. Materials and Methods

A systematic search of the literature was carried out of the following electronic databases: MEDLINE/PubMed (1966 to April 2020), EMBASE (1980 to April 2020), and the Cochrane Central Register of Controlled Trials (CENTRAL) from The Cochrane Library (2020, Issue 4) on the 29 July 2020. The search strategy for this review was constructed for each database by using a combination of medical subject headings (MeSH) and free-text terms.

Reference lists of selected articles were also examined to identify relevant studies that were not identified in the database searches. In addition, the World Health Organization International Clinical Trials Registry, ClinicalTrials.gov, ISRCTN Register and PROSPERO were searched to identify ongoing and unpublished studies.

### 2.1. Study Selection

The inclusion criteria were studies describing and evaluating the strength of association of risk factors within ESCC. All randomized controlled trials (RCTs) and observational studies, adult only studies, human studies, English studies, and studies where the patients had ESCC were included.

The exclusion criteria were pediatric studies, non-human studies, non-English studies, non-ESCC studies, case series, case reports, editorials, conference abstracts and opinions.

One author (MT) independently reviewed all studies identified by the search strategy. After removing duplicates, the titles and abstracts of the studies were screened for inclusion using Covidence software [1]. Where there was uncertainty from the study abstract, the full paper was assessed for relevance.

### 2.2. Data Extraction

Two authors (MT, SC) independently extracted data from the included studies using an electronic data extraction spreadsheet. Disagreements were resolved through discussion and where consensus could not be reached, a third independent author (SRM), was consulted.

### 2.3. Outcome Measures

The primary outcome was identifying risk factors associated with ESCC. Risk factors were divided into seven subcategories: genetic, dietary and nutrition, gastric atrophy, infection and microbiome, metabolic, epidemiological and environmental, and other risk factors.

## 3. Results and Discussion

### 3.1. Study Selection

**Figure 1:**
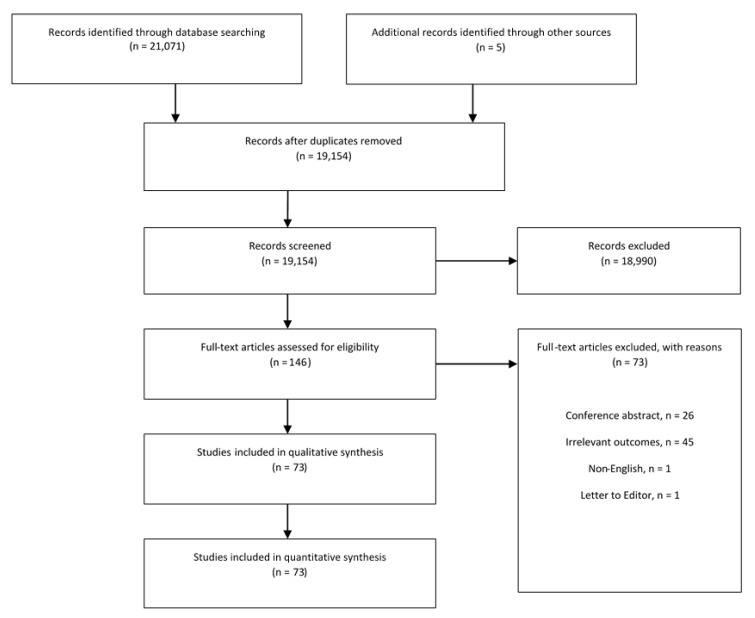
PRISMA Flowchart.

The literature search identified 21,071 studies. After removing duplicates, 19,154 abstracts were assessed for eligibility. Following abstract screening, 18,990 studies were excluded based on title and abstract relevance. Of the remaining 146 articles, 73 studies met the inclusion criteria. PRISMA flowchart summarizing the search strategy is demonstrated in Figure 1. All included studies were observational studies. These studies were divided into 22 studies investigating genetic factors, 19 studies investigating dietary and nutritional factors, 5 investigating gastric atrophy, 5 infection and microbiome, 5 metabolic factors, 4 epidemiological and environmental factors, 11 other risk factors and 2 ongoing studies. A summary of the identified risk factors associated with the development of ESCC is presented in Table 1.

### 3.2. Genetic Factors

#### 3.2.1. Candidate Genes in Tumorigenesis

The role of genetic factors in the development and prognosis of ESCC is an area that has been widely studied, but yet remains rather limited in its understanding. Unlike other malignancies of the GI tract such as colorectal cancer, ESCC does not have a well-established tumorigenesis pathway. However, there are many candidate genes, including silencing mutations of the p53 gene (Tp53). Tp53 is a tumor suppressor gene located at chromosome 17q and is implicated in the early pre-malignant processes of ESCC along with several other cancers. Alterations in p53 expression are often elevated, even in benign conditions such as esophagitis, which later leads to overt malignancy in the minority of patients. In other studies, a direct comparison of p53 expression in histologically cancerous and normal esophageal mucosa was higher in the former [2,3]. Changes to p53 protein expression is also higher in patients predisposed to other risk factors. There was a dose-dependent relationship between the frequency of mutations and exposure to specific risk factors [4]. A higher frequency of p53 mutations is reported in patients with confirmed cancerous lesions and alcohol, smoking and a high oral intake of nitrosamine compounds found in chilies, spiced foods, and hot tea [5]. Fagundes et al. showed that in p53 expression was affected in esophageal cancers of increasing severity amongst smokers and alcohol users, and changes were evident even in subjects who were asymptomatic or had normal mucosa as a response to tissue injury upon exposure to smoke or alcohol [6]. Similarly, the FHIT (fragile histidine triad) gene is a TSG located at chromosome 3p14.2 and regulates apoptosis and cell cycle arrest in a p53-independent manner. Studies on other aero-digestive cancers have reported FHIT as an exogenous target of carcinogens including tobacco and asbestos. In ESCC, Morito et al. showed a higher loss of Fhit expression (92%) in patients who were heavy users of tobacco and alcohol compared to light users (38%) [7]. This suggests that p53 and p53-independent genetic mutations might be an initial step in the interaction between lifestyle and genetic factors to mediate the development of ESCC.

#### 3.2.2. Loss of Heterozygosity

Loss of heterozygosity (LOH) is a common genetic event in cancer development. It encompasses a reduction in the number of copies of a chromosome, deletion of part of a chromosome, and homologous recombination events. LOH is strongly associated with the loss of the wild-type allele in patients who carry a germline mutation and have an inherited cancer syndrome. In esophageal cancer, studies have identified certain chromosomal regions that have exhibited LOH. For example, in a small study of 11 subjects, Hu et al. carried out genome wide scans using 366 microsatellite markers and identified 14 chromosomal regions with high frequency LOH exceeding 75% [8,9]. Allelic loss on chromosomes 2q, 3p, 4p, 4q, 5p, 5q, 6q, 8p, 9p, 9q, 11p, 13q, 14q, 15q, 17p, 17q, and 18q has been reported previously in esophageal cancer [10]. Allelic loss on chromosome 17p has been reported frequently in esophageal squamous cell carcinoma, as in many human tumors, and generally encompasses the p53 locus at 17p13 [11]. Tumor LOH frequency was significantly higher in cases with a positive family history than in those who were family-history-negative. Allelic loss is a clinically relevant genetic aberration as a prognostic marker. For example, allelic loss at D6S1027 (6q21) and D9S910 (9q22.3–q31) was more frequent in tumors that had metastasized, so there may be a role for these genes in modulating the metastatic process [9]. Meanwhile, LOH at locus D4S2361 (4q21.3–q22) was inversely associated with tumor grade. Hence, a better appreciation of LOH and linking them to clinical and pathologic tumor characteristics is required.

#### 3.2.3. ESCC in Genetic Syndromes

Malignancies are a motif in many genetic syndromes, including esophageal cancer [12]. For example, type A tylosis (focal nonepidermolytic palmoplantar keratoderma) is associated with a high risk of squamous cell esophageal cancer (up to 95% by age 65) in three extensive pedigrees. Tylosis esophageal cancer (TOC) is an autosomal dominant condition that has been mapped to a small section of chromosomal region 17q25. Blaydon et al. show that gain-of-function mutations in RHBDF2 gene leads to sustained EGFR signalling within the cells, which in turn, leads to a hyperproliferative epithelium and dysregulated wound healing seen in ESCC [13]. Developing chemotherapies targeted to RHBDF2-regulated pathways could be more effective in reducing the hyperproliferative and invasive aspects of esophageal cancers.

#### 3.2.4. Genetic Polymorphisms

The influence of genetic factors is not only limited to the development of ESCC, but also to how the cancer is handled by the body. One aspect of this involves genetic polymorphisms, which account for variation in cancer outcomes between individuals. For example, polymorphisms of LPM2-60 and LMP7-145 have been shown to generate functional alterations in how tumor antigens are processed in colorectal and gastric cancers [14]. Similar findings are also available for ESCC [15]. For example, low molecular mass protein (LMP) genes perform a critical role in the foreign antigen processing machine via the major histocompatibility complex-I (MHC-I) complex CD8+ cytotoxic T lymphocytes (CTL) pathway. The body relies on MHC-I-restricted T cells to eliminate cancer cells. In their study, Wu et al. showed that certain polymorphisms of LMP proteins (rs17587 and rs2071543 polymorphisms) were associated with an increased cancer risk in the recessive and homozygote models [16]. Similarly, in another study, a role was shown for polymorphisms of enzymes involved in phase I metabolism but not phase II metabolism. In this study, an increased risk was observed in the subjects who harbored variant genotype of CYP1A14, and the risk was further enhanced in smokers, adobe dwellers, and biomass fuel users [17]. This shows that certain polymorphisms may have a greater role in the context of exposure to other risk factors as well.

#### 3.2.5. Epigenetic Mechanisms in ESCC

DNA methylation is a main form of epigenetic inheritance and is often used as a biomarker for tumor classification, diagnosis, and prediction of metastasis or recurrence. An increase in the methylation of CpG islands in the promoter region of tumor suppressor genes (TSGs) can reduce their transcription and thus the function of TSGs. Such a phenomenon is termed epigenetic silencing and is a common molecular aberration in ESCC [18]. Paired boxed gene 1 (PAX1) and zinc finger protein 582 (ZNF582) are two tumor suppressor genes. For example, in one study, Huang et al. reported that the DNA methylation levels and frequencies of PAX1 and ZNF582 genes were markedly higher in ESCC tumor tissues compared to those in paracancerous tissues and showed a relatively good sensitivity and specificity for the detection of ESCC tumors [19]. Similarly, NAT2 is involved in the metabolism of aromatic amines, a major class of tobacco smoke carcinogens, and variant alleles in NAT2 result in slow clearance of aromatic amines [20]. While polymorphisms of the NAT2 genes did not show a relationship with ESCC risk, slow acetylators of NAT2 may be at a higher risk of ESCC if they are also exposed to other factors such as smoking.

Micro RNAs (miRNA) are small, single stranded non-coding genetic sequences with functions in RNA silencing and post-transcriptional modulation of genetic expression. In tumorigenesis, oncogenic miRNAs are often upregulated, while tumor-suppressing miRNAs are usually downregulated. For example, miR-508 inhibits several phosphatases such as PTEN in the PI3K–AKT pathway and results in metastasis of ESCC cells [21]. The over-expression of oncogenic miRNAs can also cause downregulate immune checkpoint inhibition, and thus help cancer cells evade the immune system. In contrast, miR-143-3p is a tumor suppressor that can decrease cellular proliferation and prevents metastasis but is usually downregulated in ESCC cells [22]. The deactivation of miR-29c often results in resistance to chemotherapy and radiotherapy [23]. As such, miRNAs may not only play a role in the development of ESCC but also in the cancer’s response to current treatments.

### 3.3. Dietary and Nutritional Factors

Dietary habits have been implicated in the development of ESCC. 19 studies have showed that a ‘healthy diet’ may be protective against ESCC whereas a more ‘western diet’ may increase the risk.

One of the main components of a ‘healthy diet’ is intake of vegetables and fruits. Seven studies have shown that reduced vegetable and fruit intake along with a low fiber diet increases the risk of ESCC [24,25,26,27,28,29,30]. In addition to this, Ostadrahimi et al. showed that an increase of intake of Cadmium, found in cereals and vegetables, has been found to be protective against ESCC [31]. Chen et al. demonstrated a reduction in the risk of ESCC with increase in raw onion and garlic intake [32].

Cheng et al. reported an increased risk of ESCC with a high intake of salted and pickled food [25]. Consumption of forms of smokeless tobacco including areca nut, betel quid, and oral snuff have been identified as risk factors of ESCC [32,33].

Regarding a ‘western diet’ that is mainly meat and fat based, four studies have shown an association between an increase in meat and dietary fat intake with an increased risk of ESCC [26,30,34,35]. This association has been found mainly with an increase in red meat intake. This includes processed, salted, and cured meat [34]. This may be due to an increased intake of heterocyclic amines, which are found on the surface of meats cooked at high temperature. Repeated reuse of oil while cooking has been identified as an independent risk factor [36,37]. Interestingly, Bahmanyar et al. demonstrated a reduced risk of ESCC with an increased intake of poultry and fish [29]. Ibiebele et al. demonstrated a reduced risk of ESCC in a ‘pasta and pizza’ based diet [30].

Reduced dietary intake of nutrients and vitamins have been associated with an increased risk of ESCC [24,38]. The main nutrients and vitamins identified in two studies to be risk factors were reduced intake of vitamin A, C, and E, zinc, riboflavin, selenium, and antioxidants especially folate. Nutrition deficiencies increase risk of ESCC by enhancing sensitivity to the effects of other environmental or genetic risk factors by impairing DNA repair and by altering metabolism of carcinogens [24].

Nutrition indices have also been shown to be predictors of risk in ESCC, namely, glycemic index (GI), glycemic load (GL), and dietary inflammation index (DII) in three studies [39,40,41]. GI is a ranking of carbohydrate-containing foods consumed in isoglucidic amounts that is based on their post prandial glucose response compared with standard food [40]. Since quantity and quality of ingested carbohydrates influence the post prandial glycemic response, GL was developed. GL is the product of the GI value and the total carbohydrate content of the portion of food ingested [39]. Increased dietary sugar intake, GI, and GL have been linked to development of ESCC [39,40]. This is hypothesized to be due to the modulation of the insulin-like growth factors and inhibition of apoptosis in Esophageal carcinoma cells [39]. DII is a literature-based composite scoring system that was developed to reflect the potential inflammatory effects of diet. Lu et al. reported that a higher DII score was associated with an increased risk of ESCC and thus concluded that diet-related inflammation may contribute to the etiology of ESCC [41].

Sun et al. reported that an increase in consumption of low-quality water was found to increase the risk of ESCC [42]. Furthermore, ingestion of hot foods and beverages has consistently been found to be a risk factor likely secondary to thermal damage [25,26,27,36].

### 3.4. Gastric Atrophy

Early cohort studies observed that patients not exposed to the classic risk factors such as alcohol, smoking, or processed foods still developed esophageal cancer, suggesting the possibility of other non-classical factors. This was promptly followed by work that linked gastric atrophy to cytotoxin-associated gene A (CagA)-positive Helicobacter pylori (H Pylori) and autoimmune conditions such as pernicious anemia, and subsequently gastric atrophy to ESCC [43]. In one case-controlled study of Japanese patients, although there was variation in the H pylori infection rates, gastric atrophy was more prevalent among ESCC patients compared with controls and was found to be an independent risk factor [44,45]. Such observations remain consistent in other populations, as evident from cohort studies carried out in Sweden, Russia, and Serbia [46,47]. It is possible gastric atrophy reduces the normal secretory function of the stomach, and the resulting achlorhydria forms a biome that favors bacterial overgrowth that can subsequently lead to endogenous nitrosamine formation, which can reach the esophagus during reflux of stomach contents after metabolic activation by p450 enzymes present into the middle and distal esophageal mucosa. A similar mechanism was also proposed for how autoimmune conditions increases the risk of ESCC, given that gastric atrophy is a consistent macroscopic feature of the stomach in pernicious anemia [47]. Besides autoimmune and infectious agents, gastric atrophy is also the consequence from exposure to lifestyle-based risk factors such as smoking and alcohol.

### 3.5. Infection and Microbiome

The role of infectious agents as a risk factor for ESCC is poorly understood, unlike adenocarcinoma of the esophagus where H Pylori is a well-established causative agent. One way to approach this would be to classify causative pathogens as part of the internal human microbiome, and external agents in the environment that infect patients. Typically, Streptococci are the dominant microorganisms found in the esophagus, although this is altered in disease, including premalignant conditions such as esophagitis [48]. For example, Chen et al. showed that patients with ESCC had a reduced microbial diversity and decreased levels of particular species of bacteria, including Lautropia, Bulleidia, Catonella, Corynebacterium, Moryella, Peptococcus, and Cardiobacterium [49]. The most commonly implicated agent is the human papilloma virus (HPV), especially high-risk strains such as types 16 and 18 [50]. In one meta-analysis, the risk of ESCC was increased by 4x in patients infected with the HPV 16 [51]. Like in cervical cancer, the virus integrates itself into the host genome to express oncoproteins such as E6 and E7. The E6 protein promotes cell proliferation by stimulating degradation of the p53 protein, while E7 disrupts the interaction between Rb and E2F, resulting in the release of E2F factors in their transcriptionally active forms. Together, both proteins lead to increased cellular proliferation and hence ESCC. In sub-Saharan Africa, the burden of the human immunodeficiency virus (HIV) is high and often co-exists with HPV infection. One case-controlled study showed that HIV infection was an independent risk factor for ESCC, and the effect was seen regardless of HPV status [52].

### 3.6. Metabolic Factors

Five studies focused on mechanistic pathways and end metabolites in ESCC identified pathways of interest, namely: Prostaglandin E2 (PGE_2_), acetaldehyde, and retinoid metabolism [53,54,55,56]. PGE_2_ plays a central role in the Sammon and Alderson model of esophageal carcinogenesis described in 1998 [57]. Pink et al. explored the role of PGE_2_ in ESCC carcinogenesis [53]. Their results suggest that increased esophageal and gastric fluid PGE_2_ has a direct stimulatory effect on cell division. This result points to a potential positive feedback loop in which PGE_2_ stimulates COX2 levels, leading to further metabolism of maize-derived linoleic acid into PGE_2_ leading to further stimulation of cell division. The direct mitogenic action of PGE_2_ supports the hypothesis that increased PGE_2_ in gastric fluid may be a risk factor for ESCC.

Aldehyde dehydrogenase 2 (ALDH2) deficiency is known to be an independent risk factor of early ESCC. This association was mainly seen in hypochlorhydric patients. This raises the possibility that acetaldehyde metabolism in an anacidic stomach may be involved, at least in some capacity, in the ALDH2 related risk to ESCC [54,55]. Alterations in retinoic acid receptor mediated gene transcription and retinoid metabolism have been another focus of research. These have been proposed to be a risk factor of ESCC. Diminished abundance of retinoic acid receptors through a ‘functional’ down-regulation of mRNA expression is associated with an increased risk of ESCC [56].

### 3.7. Epidemiological and Environmental Factors

ESCC rates vary greatly between and within countries. Geographical and environmental factors have been identified as possible reasons for this. Geographical high-risk areas have been categorized into two bands: the central Asian esophageal cancer belt extending across central Asia from the Caspian Sea to northern China, and the second band extending from Eastern to Southern Africa [58,59].

Two studies on environmental risk factors mainly hypothesized that trace element imbalance in the soil may be a risk factor of ESCC [59,60]. The soil in high-risk areas in South Africa was found to have lower concentrations of manganese, iron, magnesium, and sodium. These areas are known for having eroded sedimentary soils termed ‘Beaufort Soils’ [60]. Similar findings were noted in the soil along the southern shores of the Caspian Sea in Iran, a country with a high incidence and prevalence of ESCC. Low levels of minerals including boron, molybdenum, zinc, and copper, whereas levels of heavy metals such as lead tended to be excessive. Interestingly, comparative studies of soils in Africa and Iran, high-risk regions for ESCC, revealed marked similarities. Results confirmed association of risk of ESCC with high potential soils, high organic matter contents, and low subsoil pH values. Soils in both regions had low manganese [59].

The primary source of fuel in poorer communities in Africa, Asia, and South America—high-risk regions of ESCC—is solid biomass fuels from wood, charcoal, coal, dung, and crop residues. A systematic review and meta-analysis of 16 case-control studies concluded that biomass fuel is associated with significantly higher risks of ESCC [61]. Polycyclic aromatic hydrocarbons are the major component of biomass fuels which has independently been associated with an increased risk of ESCC [58].

### 3.8. Other Risk Factors

There are 11 further studies that have dissected other less common risk factors for the development of ESCC. Ionizing radiation has been postulated as a causative factor, although the evidence for this is not concrete. In the United Kingdom, it is estimated that less than 1% of esophageal cancer is linked to radiation, and ESCC accounts for an even smaller percentage of these cases. Broadly, sources of radiation can be classified as iatrogenic and non-iatrogenic, with the latter being predominantly due to occupational reasons. For example, in patients who received radiotherapy for treatment of breast cancer, the five year incidence of ESCC in the upper and middle thirds of the esophagus was increased [62,63]. Thus, therapeutic uses of irradiation at high doses over a targeted area can be a significant worrying factor. On the contrary, there is little evidence that exposure to ionizing radiation in the natural environment or at work (assuming normal precautions) is a risk factor. Interestingly, there is epidemiological evidence that geographic distributions for breast, colon, ovary, and prostate cancers are related inversely to solar radiation. A similar study proved that this relationship between mortality and ultraviolet B (UVB) exposure also exists for ESCC [64]. The pathways that explain how radiation leads to ESCC—by inducing genetic alterations outlined above—are similar to how it is a useful and an inevitable treatment modality.

Other risk factors that play a minor role include caustic injury to the esophagus (alkali/chemical burns), head and neck tumors, previous endoscopic resection of ESCC and achalasia. Achalasia is a relatively rare condition with an annual incidence rate of 0.5–1.2 per 100,000 individuals, but it is associated with a higher risk of ESCC [65]. For example, in one study, Chino et al. demonstrated that achalasia, despite being a benign disease, led to premalignant lesions that progressed to ESCC in the long-term [66]. In the samples they studied, early stage ESCC was evident in most cases and advanced cancers invading the adventitia layer—in some instances up to 20 years after initial diagnosis of achalasia. The macroscopic findings concurred with molecular analysis which showed mutations in p53, p21, p16, and EGFR. One presumed mechanism for this is that achalasia leads to stasis of food and subsequent hyperplastic esophagitis that progresses to ESCC via the dysplasia-carcinoma sequence outlined above.

Other studies have scrutinized the incidence of head and neck cancers in patients who also developed ESCC, and attributed this association to the shared lifestyle risk factor of excess smoking and alcohol use [67]. In patients with head and neck cancer, the estimated excess risk of ESCC is 21 when expressed as a standardized incidence ratio [68]. Specifically, this risk is highest for patients with hypopharyngeal and oropharyngeal cancers, followed by oral cavity, laryngeal, or nasopharyngeal cancers [69]. Patients also have a higher risk of metachronous esophageal cancer after endoscopic resection of ESCC [70]. Exposure to caustic agents (such as alkalis or hazardous chemicals) is another risk factor but with a lesser incidence. These agents not only damage the esophageal mucosa but also induce an inflammatory process [71]. Long standing esophagitis eventually progresses to ESCC. The risk of ESCC due to esophagitis caused by corrosive agents has been reported at 1000–3000 times higher than in the general population [72].

### 3.9. Ongoing Studies

The literature search identified two ongoing studies. Both study designs are case-control studies. One study is based in Malawi and aims to investigate multiple risk factors for ESCC, including HIV infection, polycyclic aromatic hydrocarbons exposure, dietary factors including fumonisin and selenium, scalding hot beverages and foods, and tobacco and alcohol consumption. The study is due to complete in December 2021 and aims to recruit 600 participants [73]. The other study is based in Western Kenya and aims to investigate risk factors including lifestyle, habits, and diet. The study completed in April 2020 and enrolled 372 participants. Results are yet to be published [74].

## 4. Conclusions

This review highlights current risk factors of ESCC. Factors identified mainly focused around seven subcategories: genetic, dietary and nutrition, gastric atrophy, infection and microbiome, metabolic, epidemiological and environmental, and other risk factors. These risk factors were explored, and explanations dissected. Most studies focused on investigating genetic, dietary, and nutritional factors, whereas this review identified other potential risk factors that have yet to be fully explored. Furthermore, there is a lack of literature on the association of these risk factors with tumor factors and disease prognosis. Further research to validate these results and their effects on tumor biology is required.

## Figures and Tables

**Table 1:** Summary of identified risk factors associated with the development of ESCC.

Subcategory	Risk Factor	Mechanistic Pathway
Genetic Factors	Genetic mutations	Mutations in tumor suppressor genes p53 and Fhit leads to less cell apoptosis. Mutations in oncogenes like EGFR leads to hyperproliferative squamous cell epithelium
Genetic Factors	Genetic polymorphisms	Polymorphisms of low molecular mass proteins (LMP) genes leads to defective antigen processing thus T cells cannot eliminate cancer cells
Genetic Factors	Epigenetic mechanisms	miR-508 inhibits several phosphatases such as PTEN in the PI3K–AKT pathway. miR-143-3p, which prevents metastasis, is downregulated in ESCC
Dietary and Nutritional Factors	Reduced vegetable, fruit, and fiber intake	-
Dietary and Nutritional Factors	Reduced Cadmium	-
Dietary and Nutritional Factors	Increased salted and pickled food	-
Dietary and Nutritional Factors	Smokeless tobacco intake	-
Dietary and Nutritional Factors	Increased red meat including processed, salted, and cured meat	Increased intake of heterocyclic amines
Dietary and Nutritional Factors	Repeated reuse of oil while cooking	-
Dietary and Nutritional Factors	Reduced vitamins A, C, E, zinc, riboflavin, selenium, and antioxidants especially folate	Enhances sensitivity to the effects of other environmental or genetic risk factors by impairing DNA repair and altering metabolism of carcinogens
Dietary and Nutritional Factors	Increased glycemic index (GI), glycemic load (GL) and dietary inflammation index (DII) diets	Modulation of the insulin-like growth factors and inhibition of apoptosis in esophageal carcinoma cells
Dietary and Nutritional Factors	Increased consumption of low-quality water	-
Dietary and Nutritional Factors	Ingestion of hot foods and beverages	Likely secondary to thermal damage
Gastric Atrophy	Atrophy of the gastric mucosa	Atrophy causes achlorhydria which causes bacterial overgrowth and so form nitrosamines which can reflux into esophagus and cause ESCC
Infection and Microbiome	HPV and HIV infections	HPV integrates into host genome leading to E6 and E7 expression. E6 promotes cell proliferation by stimulating degradation of the p53 protein. E7 disrupts the interaction between Rb and elongation 2 factors (E2Fs)
Metabolic Factors	Increased esophageal and gastric fluid PGE_2_	Direct stimulatory effect on cell division. Points to potential positive feedback loop which PGE_2_ stimulates COX2 levels, leading to further metabolism of linoleic acid into PGE_2_ leading to further stimulation of cell division
Metabolic Factors	Aldehyde dehydrogenase 2 (ALDH2) deficiency	Mainly seen in anacidic stomach/hypochlorhydric patients
Metabolic Factors	Alterations in retinoic acid receptor mediated gene transcription and retinoid metabolism	Diminished abundance of retinoic acid receptors through a ‘functional’ down-regulation of mRNA expression
Epidemiological and Environmental Factors	Geographical high-risk areas—Central Asian Esophageal cancer belt and Eastern to Southern Africa band	-
Epidemiological and Environmental Factors	Soil trace element imbalance	-
Epidemiological and Environmental Factors	Increase in use of biomass fuels	Increase in polycyclic aromatic hydrocarbons
Other Risk Factors	Exposure to ionizing radiation	Induces genetic alterations
Other Risk Factors	Achalasia	Stasis of food leading to subsequent hyperplastic esophagitis that progresses to ESCC via the dysplasia-carcinoma sequence
Other Risk Factors	Head and neck cancers	Possibly attributed to the shared lifestyle risk factor of excess smoking and alcohol use
Other Risk Factors	Previous endoscopic resection of ESCC	-
Other Risk Factors	Exposure to caustic agents	Damages the esophageal mucosa and induces an inflammatory process leading to long standing esophagitis which eventually progresses to ESCC
